# Bathymetry Determination via X-Band Radar Data: A New Strategy and Numerical Results

**DOI:** 10.3390/s100706522

**Published:** 2010-07-06

**Authors:** Francesco Serafino, Claudio Lugni, Jose Carlos Nieto Borge, Virginia Zamparelli, Francesco Soldovieri

**Affiliations:** 1 Institute for Electromagnetic Sensing of the Environment, National Research Council, Via Diocleziano 328, Napoli, I-80124 Italy; E-Mails: serafino.f@irea.cnr.it (F.S.), zamparelli.v@irea.cnr.it (V.Z.); 2 INSEAN, the Italian Ship Model Basin, Department of Seakeeping and Maneuverability, Via di Vallerano, 139, Roma, I-00128 Italy; E-Mail: c.lugni@insean.it; 3 Department of Signal Theory and Communications, University of Alcala, Alcala de Henares, Spain; E-Mail: josecarlos.nieto@uah.es

**Keywords:** sea-state monitoring, X-band Radar images, water-depth estimation

## Abstract

This work deals with the question of sea state monitoring using marine X-band radar images and focuses its attention on the problem of sea depth estimation. We present and discuss a technique to estimate bathymetry by exploiting the dispersion relation for surface gravity waves. This estimation technique is based on the correlation between the measured and the theoretical sea wave spectra and a simple analysis of the approach is performed through test cases with synthetic data. More in detail, the reliability of the estimate technique is verified through simulated data sets that are concerned with different values of bathymetry and surface currents for two types of sea spectrum: JONSWAP and Pierson-Moskowitz. The results show how the estimated bathymetry is fairly accurate for low depth values, while the estimate is less accurate as the bathymetry increases, due to a less significant role of the bathymetry on the sea surface waves as the water depth increases.

## 1. Introduction

Sea state monitoring using obtained marine radar data is of timely interest due to the fact that X-band radar systems provide the opportunity to scan the sea surface with high temporal and spatial resolution [1–6]. This possibility arises from the fact that the backscattering from the sea is captured by the marine radar ranging from some kilometers up to few tens of kilometers from the observation platform depending on the peak radiated power.

These radar signatures are considered clutter when the radar is exploited for the usual aim of the navigation control. Conversely, these radar signatures can be processed to achieve information about sea state conditions, resulting in a useful tool for regular monitoring. The intensity of clutter depends on the wind and sea state [5] and the minimum sea wave height detectable by the radar is some tens of centimeters.

The backscattering by the sea arises due to the Bragg resonance [7] of ocean waves of wavelengths similar to those of the transmitted electromagnetic waves. In particular, the longer waves modulate the backscattering phenomenon and thus they become visible in the “radar” images. More in detail, the electromagnetic scattering modulation arises due to three mechanisms such as: hydrodynamic modulation (HM), tilt modulation (TM), and shadowing (SH) [8–10].

As a result, the radar image is not a direct representation of the sea state and thus a reconstruction procedure is needed. In general, data processing is cast as an inversion problem where, starting from a time series of spatial radar images collected at different time-instants, one aims at determining the elevation *η(x,y,t)* of the sea surface meant as a function of two spatial variables (related to the area illuminated by the radar) and of the time.

In this paper we focus the attention to the problem of the determination of the sea depth starting from the images collected by an X-band radar system. This problem has significant practical motivations since coastal monitoring of the sea state, and in particular changes in water depth near the coast, is a topic of timely and great interest. In fact, the possibility of continuously measuring the evolution of sea state and bathymetry represents a key point for many applications such as: coastal erosion; control of coastal areas affected by the anomalous wave hazards; support to navigation in zones close to ports and coasts.

This problem has been already tackled in the literature in [11], where it was shown that in the case of sea depths smaller than approximately 10 meters, the variations in the wave period will have little effect on the wave speed. This suggested that in very shallow water, the wave velocity can be measured with the greatest accuracy, since some inaccuracy in the wave period measurement can be tolerated; this provides the basis of a procedure for the estimation of shallow water bathymetry. In particular, the procedure exploits the dispersion relation by measuring the wave speed via the cross-correlation between subsequent images to estimate the motion of the wave. However, this technique suffer two drawbacks. First, as the water becomes shallower, the nonlinearities in wave behaviour are more and more significant, thus making the linear theory accounted for by the gravity dispersion relation invalid; this causes an overestimation of the depth. Secondly, the spatial resolution achieved by the technique is limited, and, more important, the effect of the surface current(s) is neglected in the reconstruction.

The above drawbacks have been overcome in [12], where the near surface currents are accounted for and modifications are introduced to take into account the nonlinearities not modelled by the dispersion equation. The effectiveness of the technique was tested by comparison with measurements with buoys; however, the main constraint of the technique is the necessity to have two buoy measurements in order to estimate the parameters necessary for the inversion of the dispersion relation.

In [13] a method similar to that described by Bell [11] is presented; it is based on the determination of the wave frequency and corresponding wave number. The method exploits the dependency of the sea depth on the local wave-number through the dispersion relation and neglects the surface current(s). In particular, the wave-number for a fixed frequency at each location is measured by determining the local phase gradient, after the bathymetry is determined by using the dispersion relation. The limitations of this technique are that it assumes monochromatic waves and, as mentioned, it neglects any near surface currents.

Here, we address the problem of sea depth estimation by exploiting the technique already proposed in [14,15] where the procedure was set up for the estimation of the sea surface currents. The presented procedure strategy is based on the determination of the water depth as the quantity that globally maximizes the “correlation” between the measured sea wave spectrum and the characteristic function having a support given by the locus of points of the dispersion relation evaluated for different values of the sea depth.

In principle the approach presented here is able to simultaneously determine both the surface current and sea depth; however, here we focus the attention only on the problem of the water depth estimation. The reliability of this strategy is tested against synthetic data in the simplified case of the sea wave function with only a spatial variable *x* and of period *t* (2D case), assuming known the value of the sea surface current. The adoption of such a simplified case allows us to clearly analyze the effect of various parameters such as the sea depth; the presence or absence of sea surface current(s); the kind of sea wave spectrum.

Therefore, the paper is organized as follows. Section 2 is devoted to presenting briefly the data processing approach while in Section 3 the problem of the sea depth estimation is analyzed and the reconstruction strategy is presented. Section 4 deals with the numerical analysis of the proposed reconstruction strategy and finally the conclusions follow.

## 2. Data Processing Approach

**Figure 1.**
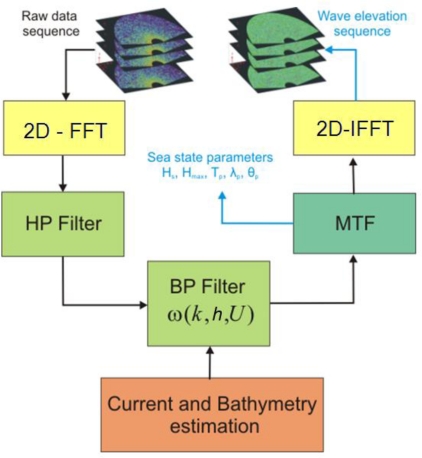
Block diagram of the inversion procedure.

This section briefly describes the solution scheme usually exploited to extract the behavior of the wave elevation in space and time from the X-band radar images. The inversion approach is here presented in a 2D (space and time) domain; therefore the sea wave elevation is a function of the time *t* and of only the spatial variable *x*. The reconstruction scheme is already described in the literature [3,14] and is schematized in the block diagram of Figure 1, where each block is detailed below.

The starting step consists in applying a 2D Fast Fourier Transform, (2D-FFT) to obtain a 2D image spectrum *F(k,ω)* from the raw data sequence. Subsequently, a High Pass (HP) filtering in the *k*-domain is applied to the image spectrum *F(k,ω)* with the aim of removing the effects due to the received signal power decay along the range (*i.e.*, *x*-direction).

In the second step, the extraction of the desired linear gravity wave components from the HP filtered image spectrum *F_1_(k,ω)* is performed. To this aim, a filtering is performed through the gravity dispersion relation that relates the wave number *k* to the pulsation *ω(k)* [1,3,16]:

(1) $$\omega (k)=\sqrt{g\left|k\right|\text{tanh}(\left|k\right|h)}+\mathrm{kU}$$

where *ω* [rad/s] and *k* [rad/m] are the pulsation and wave-number of the gravity waves, respectively; *g* is the acceleration due to gravity [m/s^2^], *U* is the sea surface current [m/s] and *h* is the sea depth [m].

The filtering procedure dictated by Equation (1) is one of the key points of the whole sea state reconstruction and an accurate knowledge of both bathymetry *h* and current *U* is necessary to build up the correct Band Pass filter [17]. In fact, inaccurate estimation of these parameters results in an incorrect spectral filtering and accordingly, an unreliable reconstruction of the sea state in the space-time domain is achieved [11–13].

The estimation of the surface current is made possible by different strategies such as the ones given in [6,10,14]. Once the current *U* and the sea depth *h* have been estimated, the band-pass (BP) filter *G*(*k,h*,*ω,U*) is built according to Equation (1) and then applied to the image spectrum *F_1_(k,ω)* so as to produce the spectrum *F̃_I_*(*k, ω*).

The successive step is to pass from the filtered radar image spectrum *F̃_I_*(*k, ω*) to the sea-wave spectrum *F_W_(k,ω)*. This step is implemented by resorting to the Modulation Transfer Function (MTF) [3]. In particular the Modulation Transfer Function (MTF) |*M* (*k*)|^2^ is applied to the filtered spectrum *F̃_I_*(*k, ω*) according to:

(2) $$F_{W}\left(k,\omega \right)=\frac{{\tilde{F}}_{I}\left(k,\omega \right)}{{\left|M(k)\right|}^{2}}$$

being |*M* (*k*)|^2^ = *k^β^*; an empirical analysis provided *β* = −1.2 [3] as a reliable estimation.

The determination of the sea wave spectrum *F_W_*(*k, ω*) allows us to determine the main parameters of the sea state; finally, also the time-space evolution of the wave height *η*(*x,t*) can be estimated by performing an inverse 2D-FFT of the function *F_W_*(*k, ω*).

## 3. Sea Depth Determination

**Figure 2.**
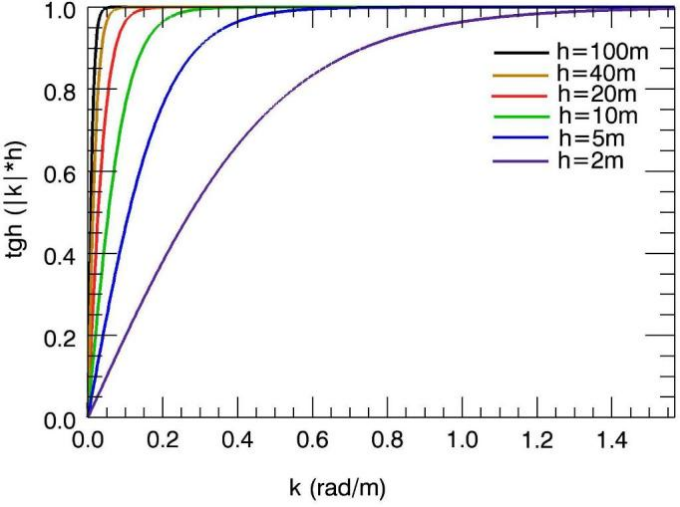
Behaviour of the tanh(|*k*|*h*) function at variance of the sea depth (sea depth values 2, 5, 10, 15, 20, 40 and 100 m).

This Section is devoted to presenting the problem of the sea depth determination and describing a strategy for the determination of such a quantity *h* starting from the X-band radar images. In this study, the sea surface current is not considered, *i.e.*, *U* = 0, so Equation (1) becomes $\omega (k)=\sqrt{g\left|k\right|\text{tanh}(\left|k\right|h)}$. In particular, we focus the analysis on the function tanh(|*k*|*h*), Figure 2 shows the behavior of the function tanh(|*k*|*h*) for six values of sea depth (h = [2, 5, 10, 20, 40 and 100 m]). According to the well known behavior of the hyperbolic tangent tanh(|*k*|*h*), for values of the argument (|*k*|*h)* ≫1, the function becomes a constant more rapidly as the sea depth increases. Differences between gravity dispersion curves are reduced with increasing water depth (as can be observed in Figure 2). This behavior has an effect on the ill-conditioning of the depth inversion problem that becomes more pronounced as the water depth increases [18]. This entails that as long as the water depth increases, the accuracy performance of any inversion strategy deteriorates.

Here, we perform the sea depth estimation by means of a technique similar to the one already presented in [14,15] for the surface current determination. This method determines the unknown sea depth quantity *h* as the one that globally maximizes the normalized scalar product (NSP) between the amplitude of the filtered image spectrum |*F_I_*(*k, ω*)| and the characteristic function *G*(*k,h*,*ω,U*) defined as:

(3) $$G\left(k,h,\omega ,U\right)=\left\{ \begin{array}{l} 1\;\text{if}\;\left|\sqrt{g\left|k\right|\text{tanh}(\left|k\right|h)}+\mathrm{kU}-\omega (k)\right|\leq \frac{\Delta \omega }{2}\\ 0\;\text{otherwise} \end{array}\right.$$

where Δ*ω* is the frequency step used to sample the sea wave spectrum.

The NSP (as function of the current components *U* and the depth *h*) is defined as:

(4) $$V\left(U,h\right)=\frac{\langle \left|F_{I}\left(k\omega \right)\right|,G\left(k,h,\omega ,U\right)\rangle }{\sqrt{P_{F}\cdot P_{G}}}$$

where < · > denotes the scalar product in the sea spectra space, and *P_F_* and *P_G_* are the power associated to the image spectrum |*F_I_* (·)| and *G*(·), respectively. The effectiveness of the proposed strategy is analyzed in the Section below by considering only the sea depth estimation problem.

## 4. Validation of the Approach by Synthetic Data

This Section aims at showing the effectiveness of the proposed strategy against synthetic data. Synthetic data have been generated using the linear theory for wave propagation in finite depth condition [19]. The long-crested wave field is computed as a linear superposition of N wave components:

(5) $$\eta (x,t)=\sum_{i=1}^{N}A(\omega_{i})\text{cos}[\omega_{i}t-k(\omega_{i})x-\varphi (\omega_{i})]$$

where *ω_i_* is the circular frequency and the amplitude *A*(*ω_i_*) is chosen according to a prescribed sea spectrum *S*(*ω*), *k*(*ω_i_*) is the wave-number which satisfies the relation dispersion for the fixed value of the sea-depth *h*; the phase shift *φ*(*ω_i_*) is randomly generated through a suitable algorithm.

In particular, fixed the sea spectrum *S*(*ω*), the amplitude *A*(*ω*) in correspondence to the generic pulsation ω is given as $A(\omega )=\sqrt{2S(\omega )\Delta \omega }$ where Δ*ω* is the constant difference between two successive frequencies.

The effect of the surface current is taken into account by reformulating the sea spectrum *S′*(*ω_e_*) as function of the encounter circular frequency *ω_e_* defined as *ω_e_* = *ω* − *kU*cos *β* with β representing the direction of the sea current with respect to the direction of propagation of the wave system; finally, *S′*(*ω_e_*) = *S*(*ω*) / |1–2*ωU* cos(*β*) / *g* |. The Equation (5) is used with *ω_e_* instead of the absolute circular frequency *ω* to generate the wave field. In the following β = 0, π is assumed for the cases at hand.

For the presented results, we have considered two theoretical models of scalar spectral density, namely the Pierson-Moskowitz sea spectrum (PM) [20] and the JONSWAP one [21].

The Pierson-Moskowitz sea spectrum is the typical parameterization of the scalar spectrum of the waves. For this spectrum an important hypothesis is made, that is if the wind blows constantly for a “long time” on a “wide area” then the waves are in equilibrium with the wind. This is the concept of a fully developed sea. “Long-time” means ten thousand wave periods and “wide area” means five thousand wavelengths.

To this assumption, the spectral density takes the following form:

(6) $$S_{\mathrm{PM}}\left(f\right)=\frac{\alpha g^{2}}{{\left(2\pi \right)}^{4}f^{5}}\text{exp}\left\{-0.74{\left(\frac{g}{2\pi \mathrm{Vf}}\right)}^{4}\right\}$$

with *α* = 8.1·10^−3^ being the Phillips constant and *V* is the wind speed, assumed equal to 19.5 m.

The second spectrum is the JONSWAP one, where JONSWAP is the acronym of “Joint North Sea Wave Project”. For the JONSWAP model it is expected that the sea continues to develop through nonlinear wave-wave interactions, even after a long time and over long distances. Therefore, the spectrum corresponds to a sea wind spectrum partially developed, *i.e.*, with an alteration in the parameterization proposed by PM. In this case, the spectral density is given by:

(7) $$S_{J}\left(f\right)=\frac{\alpha g^{2}}{{\left(2\pi \right)}^{4}f^{5}}\text{exp}\left\{-\frac{5}{4}{\left(\frac{f_{p}}{f}\right)}^{4}\right\}\gamma^{\text{exp}\left\{\frac{{\left(f-f_{p}\right)}^{2}}{2\sigma^{2}f_{p}^{2}}\right\}}$$

where $f_{p}=\frac{3.5g}{V}{\left(\frac{gF}{V^{2}}\right)}^{0.33}$ is frequency spectral peak, $\alpha =0.0766{\left(\frac{\mathrm{gF}}{V^{2}}\right)}^{-0.22}$, being *V* the wind speed, *F* the fetch length (which is the area where the wind that generated the waves is blowing) and *γ* a factor defined below. The quantity *F* (fetch length or fetch effective) is assessed by procedures that are based on knowledge of the geographic fetch. Fetch is a fundamental term in the JONSWAP spectrum, because it represents the difference compared to the Pierson-Moskowitz spectrum, and therefore accounts for the difference between a fully developed sea and a partially developed sea. The parameter *γ* is the ratio between the peak of the JONSWAP spectrum and the maximum value of the associated Pierson-Moskowitz spectrum.

Let us turn now to present the sea-depth estimation results. First, a JONSWAP sea spectrum with H_1/3_ = 3.25 m and T_0_ = 6.25 s has been generated. Here, H_1/3_ represents the significant wave height, and T_0_ the modal period associated with the prescribed spectrum. The second synthesized sea spectrum is a Pierson-Moskowitz (PM) one, with *H*_1/3_ = 3.25*m* and *T_p_* = 7.5 sec.

The main parameters exploited in the sea wave simulation are reported in Table 1. These data have been decimated so that the samples actually used at the reconstruction stage are *Nx* = 500 and *Nt* = 256 with steps of 4 m and 0.6 s, in space and time, respectively; these quantities are chosen so to simulate the observation modalities typical of a X-band radar having a work frequency of 9.3 GHz.

The effects of both the surface current and sea depth were added to the data; in particular, we have considered the sea depth values in a range [5 m, 25 m] with a step equal to 1 m and surface current values in a range [−5 m/s, 5 m/s] with a 0.5 m/s step. For each of the pair of values (*U*, *h*), the corresponding radar data have been generated by exploiting the procedure proposed in [5], where the model of the electromagnetic scattering exploits the geometrical optics approximation and the shadowing and tilt modulation are accounted for.

Finally, starting from each radar image set, the sea depth has been evaluated via the NSP procedure presented in the previous Section. The results presented below refer to the cases when the surface current is assumed known and therefore the NSP in (4) is maximized only with respect to the sea depth *h*.

**Figure 3.**
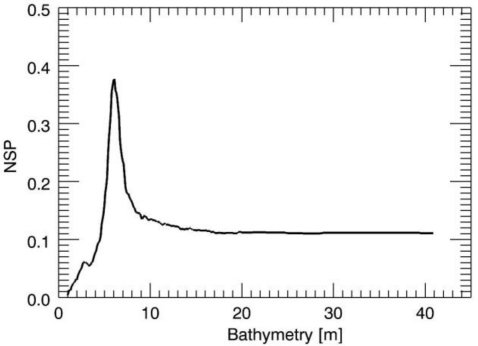
Behavior of the NSP function in the case of *h* = 6m and null surface current.

**Figure 4.**
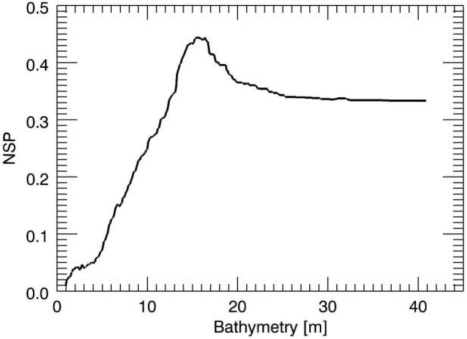
Behavior of the NSP function in the case of *h* = 15 m and null surface current.

**Figure 5.**
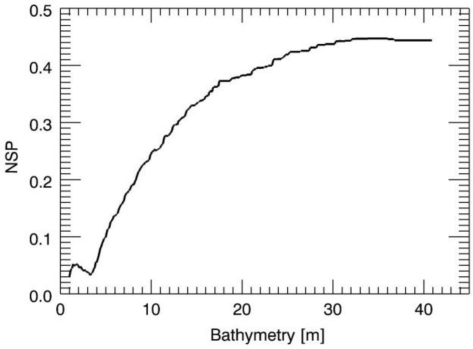
Behavior of the NSP function in the case of *h* = 30 m and null surface current.

First, we present in detail some results of the large numerical analysis. The first test case is concerned with the JONSWAP case and a true sea depth *h* = 6 m, while the true surface current is equal to zero. Figure 3 depicts the NSP function for the values of the depth in the search interval [1,40] m. The location of the maximum of the NSP function provides the correct sea depth value. The examination of Figures 3–5, allows some considerations. We note that the NSP criterion works well for the small values of the depth whereas as long as the depth increases the NSP performances deteriorate and finally for the value of *h* = 30 m, the proposed strategy fails since its accuracy is very low. This behaviour agrees well with the reasoning of the previous Section where we pointed out how the ill-conditioning of the problem [18] becomes significant for increasing values of the depth, thus affecting the performances of all reconstruction approaches used to solve the problem.

The same analysis has been performed for the sea depth values equal to 15 m and 30 m and Figures 4 and 5 depict the related NSP behaviour.

**Figure 6.**
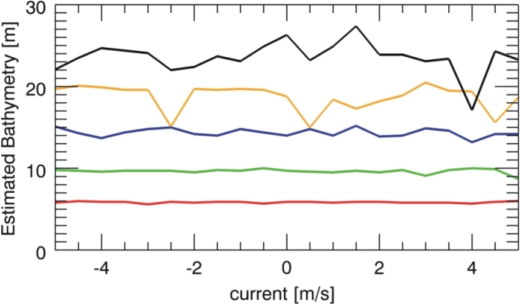
Sea depth reconstruction for the different true values equal to 5 m (red line), 10 m (green line), 15 m (blue line), 20 m (orange line), 25 m (black line), when the current varies (JONSWAP sea spectrum).

**Figure 7.**
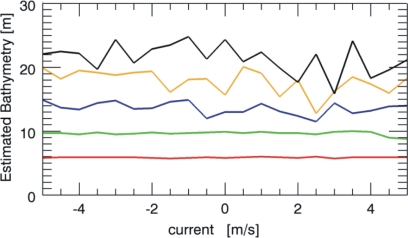
Sea depth reconstruction for the different true values equal to 5 m (red line), 10 m (green line), 15 m (blue line), 20 m (orange line), 25 m (black line), when the current varies (Pierson-Moskowitz sea spectrum).

The outcomes of the overall numerical analysis are summarised by Figures 6 and 7 that depict the reconstructed sea depth for five different values (5, 10, 15, 20, 25 m) considered in the analysis at variance of the sea surface current. As mentioned above, the results are achieved by performing the estimation of the only sea depth while assuming known accurately the surface current. Figure 6 is concerned with the JONSWAP spectrum and according to the reasoning above, we have that the estimation is satisfyingly accurate till to the value of 15 m independently of the surface current. After, the estimation also starts to deteriorate, even though it is still reliable at a depth of 25 m.

Similar performances of the estimation procedure hold also for the Pierson-Moskowitz spectrum as reported in Figure 7. Accordingly, the two figures point out how the performances of the procedure are not strongly dependent on the considered sea spectrum under investigation.

The considerations above can be summarised in Table 2, which reports the maximum error and the mean error for both the sea spectrum, for all the considered values of bathymetry in the assumed range [5 m, 25 m].

**Figure 8.**
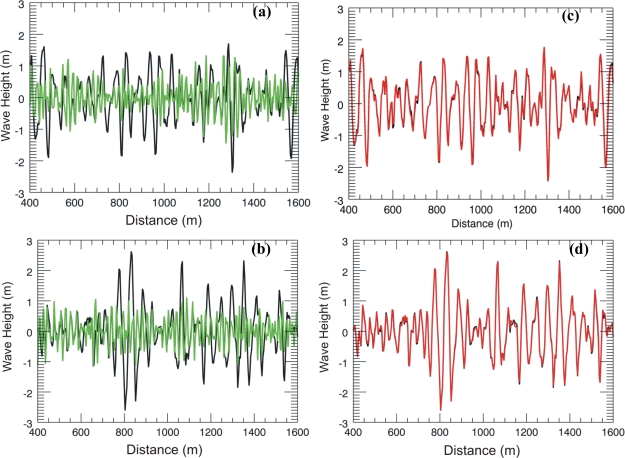
Panels (a) and (b): Comparison between the true wave height (black) and the reconstructed wave height obtained by supposing a deep water case [tanh(*kh*) = 1]. Panels (c) and (d) same as (a) and (b), but with a reconstructed wave height (red line) obtained by using the correct sea depth equal to 6 m.

Finally, Figure 8 shows the effect of the inaccuracy of the sea-depth estimation on the overall sea state monitoring for the case of a true sea depth of 6 m and null surface current. Panels 8a and 8b depict the comparison between the true wave height (black line) and the inaccurate reconstructed wave height (green line) obtained by supposing erroneously a deep water case [tanh(*kh*) = 1]. Conversely, when the sea-depth is accurately accounted for in the inversion model, a reliable wave height reconstruction is achieved, as depicted in panels 8c and 8d.

## 5. Conclusions

The paper has dealt with the problem of the sea depth estimation starting from X-band radar measurements. First, a simple analysis of the mathematical features of the problem was performed and after we have presented an estimation strategy. Then numerical analysis has provided results coherent with the theoretical expectations and pointed out how the intrinsic ill-conditioning of the problem makes it inapplicable for large values of the sea depth. In addition, the results showed that the proposed method is accurate and independent from the type of input data, which is captured by the radar and good performances of the approach were observed for a range of sea-depths up to about twenty meters. The main contribution of the work was the adoption of a “correlation” procedure to estimate the sea depth. Such a procedure has been already compared with the classical Least Square approach, largely used in literature, when we aimed at determining the sea surface current and the better performances of the correlation approach have been outlined (for such a comparison see [14])

Despite of the encouraging results, some factors have to be considered to reach a full assessment of the proposed approach. An estimation with real data is also necessary in order to analyse the effect of some limiting factors such as the presence of breaking waves in shallower water (6–10 m depth) and other non-linear wave behaviours that are not accounted for by the dispersion relation.

First, we aim at addressing one of the main factors limiting the effectiveness of proposed approach, and in general for the overall sea state monitoring, which is concerned with passage from the radar images to the sea state. This is a timely topic of significant interest that however until now has been tackled mostly on an empirical basis. The presented analysis was focused on the estimation of bathymetry considered constant in all the area seen by the radar. This assumption was necessary to conduct a preliminary study of the problem, although it represents a strong limit. Future developments will extend the analysis to the more realistic case of non-uniform bathymetry. Finally, it is noteworthy that the presented analysis and the proposed strategy hold in cases more general with respect to the radar data and are of interest in the case also of video images and other kind of sensors.

## Figures and Tables

**Table 1.** Parameters of the numerical analysis.

**Parameter**	**Value**
Time step (Δ*t*)	0.2 s
Spatial Step (Δ*x*)	2 m
Number of time steps (*N_t_*)	2,500
Number of spatial steps (*N_x_)*	1,000

**Table 2.** Estimation errors for the JONSWAP and Pierson-Moskowitz spectrum.

	**JONSWAP**		**PIERSON-MOSKOWITZ**	

**True Value**	**Max error**	**Mean Error**	**Max error**	**Mean Error**

**5.000**	0.6000	0.1927	0.6000	0.1632
**6.000**	0.4000	0.1799	0.3000	0.1543
**7.000**	0.4000	0.1988	0.5000	0.2400
**8.000**	0.6000	0.2672	0.5000	0.2645
**9.000**	0.6000	0.3147	0.7000	0.3450
**10.000**	1.3000	0.4561	1.2000	0.4477
**11.000**	0.9000	0.4191	1.7000	0.6690
**12.000**	1.7000	0.5944	2.0000	0.7656
**13.000**	2.7000	0.7807	2.2000	1.1073
**14.000**	2.9000	1.2112	3.1000	1.0151
**15.000**	1.8000	0.8050	3.5000	1.6874
**16.000**	3.5000	1.4607	3.3000	1.8299
**17.000**	4.6000	1.6897	3.0000	1.8938
**18.000**	5.20000	1.4912	5.2000	2.3053
**19.000**	6.60000	2.0715	4.7000	2.0073
**20.000**	5.00000	2.0422	7.2000	2.8224
**21.000**	8.10000	2.8181	5.7000	3.2339
**22.000**	7.80000	2.8181	5.4000	3.2171
**23.000**	5.90000	1.7681	7.6000	3.5082
**24.000**	10.4000	4.3861	6.2000	3.5211
**25.000**	7.90000	2.3711	9.1000	4.2168
